# 4,4′-Methyl­enedianilinium bis­(3-carb­oxy-4-hydroxy­benzene­sulfonate) monohydrate

**DOI:** 10.1107/S1600536808029115

**Published:** 2008-09-17

**Authors:** Guihuan Du, Zuli Liu, Qian Chu, Zhen Li, Suming Zhang

**Affiliations:** a Department of Physics, Huazhong University of Science and Technology, Wuhan 430074, People’s Republic of China; b Tongji Hospital, Huazhong University of Science and Technology, Wuhan 430070, People’s Republic of China

## Abstract

Co-crystallization of 4,4′-methyl­enediphenyl­amine (MDA) and 5-sulfosalicylic acid (5-H_2_SSA) yields the title salt, C_13_H_16_N_2_^2+^·2C_7_H_5_O_6_S^−^·H_2_O. The asymmetric unit is comprised of one dication, two anions and one water mol­ecule. In the crystal structure, the components of the salt are linked by a combination of inter­molecular O—H⋯O, N—H⋯O and weak C—H⋯O hydrogen bonds into a three-dimensional framework. In addition, two weak *π–π* inter­actions [with centroid–centroid distances of 3.8734 (15) and 3.7465 (15) Å] and one C—H*⋯π* inter­action further stabilize the crystal structure.

## Related literature

For related structures, see: Smith (2005 ▶); Smith *et al.* (2005*a* ▶,*b* ▶, 2006 ▶). For background information, see: Wang *et al.* (2008 ▶). 
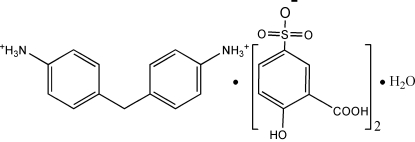


## Experimental

### 

#### Crystal data

C_13_H_16_N_2_^2+^·2C_7_H_5_O_6_S^−^·H_2_O
*M* *_r_* = 652.63
Monoclinic, 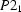

*a* = 5.8769 (1) Å
*b* = 18.8659 (3) Å
*c* = 12.9864 (2) Å
β = 94.668 (1)°
*V* = 1435.06 (4) Å^3^
*Z* = 2
Mo *K*α radiation
μ = 0.26 mm^−1^
*T* = 296 (2) K
0.40 × 0.30 × 0.04 mm

#### Data collection

Bruker SMART APEX CCD area-detector diffractometer
Absorption correction: multi-scan (*SADABS*; Sheldrick, 1996 ▶) *T*_min_ = 0.894, *T*_max_ = 0.990
16262 measured reflections
6379 independent reflections
5671 reflections with *I* > 2σ(*I*)
*R*_int_ = 0.023

#### Refinement

*R*[*F* ^2^ > 2σ(*F* ^2^)] = 0.040
*wR*(*F* ^2^) = 0.106
*S* = 1.09
6379 reflections
433 parameters
1 restraint
H atoms treated by a mixture of independent and constrained refinement
Δρ_max_ = 0.33 e Å^−3^
Δρ_min_ = −0.17 e Å^−3^
Absolute structure: Flack (1983 ▶), 3009 Friedel pairs
Flack parameter: 0.05 (6)

### 

Data collection: *SMART* (Bruker, 2007 ▶); cell refinement: *SAINT-Plus* (Bruker, 2007 ▶); data reduction: *SAINT-Plus*; program(s) used to solve structure: *SHELXS97* (Sheldrick, 2008 ▶); program(s) used to refine structure: *SHELXL97* (Sheldrick, 2008 ▶); molecular graphics: *PLATON* (Spek, 2003 ▶); software used to prepare material for publication: *PLATON*.

## Supplementary Material

Crystal structure: contains datablocks global, I. DOI: 10.1107/S1600536808029115/lh2691sup1.cif

Structure factors: contains datablocks I. DOI: 10.1107/S1600536808029115/lh2691Isup2.hkl

Additional supplementary materials:  crystallographic information; 3D view; checkCIF report

## Figures and Tables

**Table 1:** Hydrogen-bond geometry (Å, °)

*D*—H⋯*A*	*D*—H	H⋯*A*	*D*⋯*A*	*D*—H⋯*A*
N1—H1*A*⋯O4^i^	0.90 (4)	2.03 (4)	2.896 (4)	161 (3)
N1—H1*B*⋯O1^ii^	0.82 (4)	2.47 (3)	2.751 (3)	101 (3)
N1—H1*B*⋯O12	0.82 (4)	2.01 (4)	2.814 (4)	168 (3)
N1—H1*C*⋯O11^iii^	0.99 (4)	1.92 (4)	2.870 (3)	161 (3)
N2—H2*A*⋯O4^iii^	0.82 (4)	2.07 (4)	2.801 (4)	149 (3)
N2—H2*C*⋯O5	0.97 (4)	2.16 (4)	2.927 (4)	135 (3)
N2—H2*B*⋯O6	0.84 (4)	2.20 (4)	2.889 (4)	139 (3)
N2—H2*C*⋯O3^i^	0.97 (4)	2.19 (4)	2.940 (3)	134 (3)
O2—H2*D*⋯O11^iv^	0.80 (4)	1.91 (4)	2.688 (3)	164 (4)
O3—H3*A*⋯O1	0.86 (4)	1.77 (4)	2.569 (3)	153 (4)
O8—H8*A*⋯O13^v^	0.87 (5)	1.76 (5)	2.598 (4)	160 (4)
O9—H9*A*⋯O7	0.86 (5)	1.96 (4)	2.678 (3)	141 (4)
O9—H9*A*⋯O6^vi^	0.86 (5)	2.38 (4)	2.872 (3)	117 (3)
O13—H13*A*⋯O10^vii^	0.83 (8)	1.93 (8)	2.759 (4)	176 (7)
O13—H13*B*⋯O6	0.89 (7)	2.39 (7)	3.064 (4)	132 (6)
C2—H2⋯O5^viii^	0.93	2.54	3.452 (4)	168
C6—H6⋯O12	0.93	2.48	3.210 (3)	136
C12—H12⋯O9^ix^	0.93	2.55	3.428 (4)	158
C16—H16⋯*Cg*^x^	0.93	2.85	3.727 (3)	157

Symmetry codes: (i) 
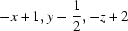
; (ii) 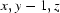; (iii) 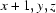; (iv) 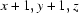; (v) 
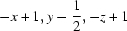
; (vi) 
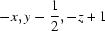
; (vii) 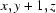; (viii) 
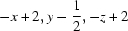
; (ix) 
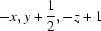
; (x) 
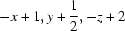
. *Cg* is the centroid of the C8–C13 ring.

**Fig. 1.**
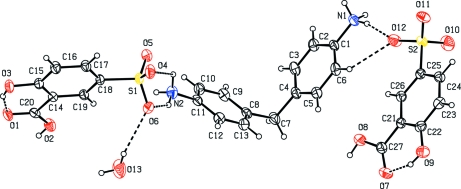
Molecular structure of (I), showing the atom-numbering scheme. Displacement ellipsoids are drawn at the 30% probability level. H-bonds are shown as dashed lines.

**Fig. 2.**
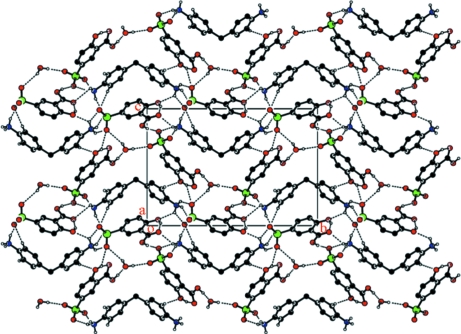
Part of the crystal structure of (I), showing the formation of the three-dimensional framework structure. Hydrogen bonds are shown as dashed lines. For the sake of clarity, H atoms not involved in the motif have been omitted from the drawing.

## supplementary crystallographic information

### Comment

In a continuation of our studies on the molecular and supra-molecular structures in organic salts formed by 5-sulfosaliyclic acid (5-H_2_SSA) and N-containing lewis bases (Wang *et al.*, 2008), we now report our findings on the title compound (Scheme I).

Two 5-HSSA^- ^anions, one 4,4'-methylenediphenylammonium dication (MDA^2+^) and one water molecules comprise the asymmetric unit of (I) (Fig. 1). As in similar analogous organic adducts which have been previuosly reported (Smith *et al.*, 2005*a*,b; Smith, 2005; Smith *et al.*, 2006), both the sulfonic H atoms are transferred to the amine N atom, yielding the title organic salt. However, the conformations of the sulfonate groups are different in the two anions. The perpendicular distances of the sulfonate O4, O5 and O6 atoms to their adjacent benzene plane are 0.585 (1), 1.263 (1) and 0.967 (1) Å, respectively. The corresponding distances are 1.456 (1), 0.844 (1) and 0.312 (1)Å for O10, O11 and O12 atoms, respectively.

In the crystal structure, the component ions are linked by a combination of O—H···O, N—H···O and C—H···O hydrogen bonds (Table 1), forming a three-dimensional network (Fig.2). An analysis using *PLATON* (Spek, 2003) showed that two *π-π* [*Cg*1···*Cg*3 = 3.8734 (15) and d_perpendicular_ = 3.522 (2) Å, symmetry code: 1 + *x*, *y*, *z*; *Cg*2···*Cg*3 = 3.7465 (15) and d_perpendicular_ = 3.526 (2), symmetry code: 1 - *x*,1/2 + *y*,1 - *z*, where *Cg*1, *Cg*2 and *Cg*3 are the centroids of the C1—C6, C8—C13 and C21—C26 benzene rings, respectively] and one C—H···*π* [*d*_H16—_*_Cg_*_2_= 2.85 Å, *d*_C16—_*_Cg_*_2_= 3.727 (3) Å, *A*_C16—H16···_*_Cg_*_2_=157°, symmetry code: 1 - *x*,1/2 + *y*,2 - *z*] interactions exist, which further consolidate the crystal structure.

### Experimental

All reagents and solvents were used as obtained without further purification. Equivalent molar amount of 4,4'-methylenediphenylamine and 5-sulfosalicylic acid dihydrate were dissolved in 95% methanol (20 ml). The mixture was stirred for 30 minutes at 300 K and then filtered. Colorless plate crystals of (I) suitable for single-crystal X-ray diffraction analysis grew at the bottom of the vessel in two weeks after slow evaporation of the solution.

### Refinement

The title compound is racemic in solution but spontaneously resolved upon crystallization. The absolute configuration of the molecules in the crystal selected was readily determined and the configuration has no chemical significance.

H atoms bonded to C atoms were positioned geometrically with C–H = 0.93 Å (aromatic), 0.97 Å (methylene) and refined in a riding mode [*U*_iso_(H) = 1.2*U*_eq_(C)]. H atoms bonded to N and O atoms were found in difference maps and the N—H and O—H distances were refined freely [the refined distances are given in Table 1; *U*_iso_(H) = 1.2*U*_eq_(N) and 1.5*U*_eq_(O), respectively].

### Crystal data

**Table:**

C_13_H_16_N_2_^2+^·2C_7_H_5_O_6_S^−^·H_2_O	*F*(000) = 680
*M**_r_* = 652.63	*D*_x_ = 1.510 Mg m^−^^3^
Monoclinic, *P*2_1_	Mo *K*α radiation, λ = 0.71073 Å
Hall symbol: P 2yb	Cell parameters from 7439 reflections
*a* = 5.8769 (1) Å	θ = 2.7–27.0°
*b* = 18.8659 (3) Å	µ = 0.26 mm^−^^1^
*c* = 12.9864 (2) Å	*T* = 296 K
β = 94.668 (1)°	Plate, colorless
*V* = 1435.06 (4) Å^3^	0.40 × 0.30 × 0.04 mm
*Z* = 2	

### Data collection

**Table:**

Bruker SMART APEX CCD area-detector diffractometer	6379 independent reflections
Radiation source: fine focus sealed Siemens Mo tube	5671 reflections with *I* > 2σ(*I*)
graphite	*R*_int_ = 0.023
0.3° wide ω exposures scans	θ_max_ = 27.5°, θ_min_ = 1.6°
Absorption correction: multi-scan (SADABS; Sheldrick, 1996)	*h* = −7→7
*T*_min_ = 0.894, *T*_max_ = 0.990	*k* = −24→24
16262 measured reflections	*l* = −16→16

### Refinement

**Table:**

Refinement on *F*^2^	Secondary atom site location: difference Fourier map
Least-squares matrix: full	Hydrogen site location: inferred from neighbouring sites
*R*[*F*^2^ > 2σ(*F*^2^)] = 0.040	H atoms treated by a mixture of independent and constrained refinement
*wR*(*F*^2^) = 0.106	*w* = 1/[σ^2^(*F*_o_^2^) + (0.0586*P*)^2^ + 0.1606*P*] where *P* = (*F*_o_^2^ + 2*F*_c_^2^)/3
*S* = 1.09	(Δ/σ)_max_ < 0.001
6379 reflections	Δρ_max_ = 0.33 e Å^−^^3^
433 parameters	Δρ_min_ = −0.17 e Å^−^^3^
1 restraint	Absolute structure: Flack (1983), 3009 Friedel pairs
Primary atom site location: structure-invariant direct methods	Flack parameter: 0.05 (6)

### Special details

**Table:**

Geometry. All e.s.d.'s (except the e.s.d. in the dihedral angle between two l.s. planes) are estimated using the full covariance matrix. The cell e.s.d.'s are taken into account individually in the estimation of e.s.d.'s in distances, angles and torsion angles; correlations between e.s.d.'s in cell parameters are only used when they are defined by crystal symmetry. An approximate (isotropic) treatment of cell e.s.d.'s is used for estimating e.s.d.'s involving l.s. planes.
Refinement. Refinement of *F*^2^ against ALL reflections. The weighted *R*-factor *wR* and goodness of fit *S* are based on *F*^2^, conventional *R*-factors *R* are based on *F*, with *F* set to zero for negative *F*^2^. The threshold expression of *F*^2^ > σ(*F*^2^) is used only for calculating *R*-factors(gt) *etc*. and is not relevant to the choice of reflections for refinement. *R*-factors based on *F*^2^ are statistically about twice as large as those based on *F*, and *R*- factors based on ALL data will be even larger.

### Fractional atomic coordinates and isotropic or equivalent isotropic displacement parameters (Å^2^)

**Table:**

	*x*	*y*	*z*	*U*_iso_*/*U*_eq_	
C1	0.9685 (4)	0.25024 (11)	0.7833 (2)	0.0379 (5)	
C2	1.1742 (5)	0.28453 (14)	0.8000 (2)	0.0466 (6)	
H2	1.2839	0.2684	0.8502	0.056*	
C3	1.2157 (5)	0.34335 (14)	0.7412 (3)	0.0490 (7)	
H3	1.3549	0.3667	0.7521	0.059*	
C4	1.0543 (5)	0.36826 (13)	0.6662 (2)	0.0430 (6)	
C5	0.8515 (5)	0.33235 (15)	0.6504 (2)	0.0491 (7)	
H5	0.7427	0.3477	0.5993	0.059*	
C6	0.8060 (5)	0.27345 (14)	0.7097 (2)	0.0477 (6)	
H6	0.6667	0.2501	0.6993	0.057*	
C7	1.0939 (6)	0.43468 (15)	0.6053 (2)	0.0537 (7)	
H7A	1.0142	0.4309	0.5371	0.064*	
H7B	1.2556	0.4397	0.5969	0.064*	
C8	1.0094 (5)	0.49994 (13)	0.6599 (2)	0.0419 (6)	
C9	1.1433 (5)	0.53092 (14)	0.7404 (2)	0.0464 (6)	
H9	1.2875	0.5125	0.7594	0.056*	
C10	1.0668 (5)	0.58854 (14)	0.7929 (2)	0.0451 (6)	
H10	1.1597	0.6096	0.8456	0.054*	
C11	0.8526 (4)	0.61432 (12)	0.7664 (2)	0.0398 (5)	
C12	0.7149 (4)	0.58484 (15)	0.6869 (2)	0.0442 (6)	
H12	0.5702	0.6031	0.6689	0.053*	
C13	0.7954 (5)	0.52752 (15)	0.6342 (2)	0.0486 (6)	
H13	0.7032	0.5073	0.5805	0.058*	
N1	0.9214 (5)	0.18836 (12)	0.8458 (2)	0.0443 (5)	
H1B	0.804 (6)	0.1679 (19)	0.824 (3)	0.053*	
H1A	0.928 (5)	0.1973 (18)	0.914 (3)	0.053*	
H1C	1.040 (6)	0.1513 (18)	0.845 (3)	0.053*	
N2	0.7665 (5)	0.67217 (14)	0.8262 (2)	0.0515 (6)	
H2A	0.861 (6)	0.703 (2)	0.841 (3)	0.062*	
H2B	0.665 (6)	0.696 (2)	0.793 (3)	0.062*	
H2C	0.692 (6)	0.6611 (19)	0.888 (3)	0.062*	
C14	0.5812 (4)	0.96279 (12)	0.95964 (18)	0.0340 (5)	
C15	0.4353 (4)	0.99410 (13)	1.02603 (19)	0.0365 (5)	
C16	0.2674 (5)	0.95376 (14)	1.0667 (2)	0.0445 (6)	
H16	0.1755	0.9739	1.1138	0.053*	
C17	0.2356 (5)	0.88431 (13)	1.0379 (2)	0.0407 (6)	
H17	0.1199	0.8579	1.0643	0.049*	
C18	0.3759 (4)	0.85330 (11)	0.96958 (19)	0.0347 (5)	
C19	0.5485 (4)	0.89143 (12)	0.93132 (19)	0.0351 (5)	
H19	0.6439	0.8701	0.8867	0.042*	
C20	0.7674 (4)	1.00540 (13)	0.91927 (19)	0.0379 (5)	
O1	0.7991 (3)	1.06703 (9)	0.94503 (16)	0.0494 (5)	
O2	0.8919 (4)	0.97200 (11)	0.85615 (17)	0.0505 (5)	
H2D	0.967 (7)	1.001 (2)	0.831 (3)	0.076*	
O3	0.4490 (4)	1.06391 (9)	1.05113 (16)	0.0493 (5)	
H3A	0.565 (7)	1.079 (2)	1.021 (3)	0.074*	
O4	0.0960 (4)	0.74792 (10)	0.94941 (17)	0.0548 (5)	
O5	0.4898 (4)	0.72016 (10)	0.99028 (16)	0.0533 (5)	
O6	0.3678 (3)	0.76210 (11)	0.82021 (14)	0.0492 (4)	
S1	0.32980 (11)	0.76440 (3)	0.92902 (5)	0.03782 (15)	
C21	0.0913 (5)	0.20452 (12)	0.48434 (19)	0.0384 (5)	
C22	−0.1231 (5)	0.17470 (14)	0.4583 (2)	0.0420 (6)	
C23	−0.1983 (4)	0.11873 (15)	0.5176 (2)	0.0440 (6)	
H23	−0.3442	0.1004	0.5028	0.053*	
C24	−0.0591 (4)	0.09056 (13)	0.5976 (2)	0.0400 (5)	
H24	−0.1094	0.0527	0.6355	0.048*	
C25	0.1583 (4)	0.11918 (13)	0.62162 (18)	0.0368 (5)	
C26	0.2297 (4)	0.17632 (13)	0.56590 (19)	0.0373 (5)	
H26	0.3726	0.1962	0.5833	0.045*	
C27	0.1761 (5)	0.26283 (15)	0.4210 (2)	0.0466 (6)	
O7	0.0906 (5)	0.27667 (13)	0.33542 (18)	0.0716 (7)	
O8	0.3540 (4)	0.29627 (12)	0.46501 (18)	0.0578 (6)	
H8A	0.407 (7)	0.328 (3)	0.424 (3)	0.087*	
O9	−0.2667 (4)	0.19688 (13)	0.37804 (16)	0.0554 (5)	
H9A	−0.206 (7)	0.231 (2)	0.348 (3)	0.083*	
O10	0.4348 (4)	0.01754 (12)	0.6798 (2)	0.0732 (7)	
O11	0.2003 (3)	0.06836 (11)	0.80492 (16)	0.0520 (5)	
O12	0.5149 (4)	0.13324 (13)	0.74758 (17)	0.0589 (5)	
S2	0.34239 (10)	0.08090 (3)	0.72033 (5)	0.03813 (15)	
O13	0.4004 (7)	0.87302 (16)	0.6497 (3)	0.0986 (11)	
H13A	0.404 (11)	0.917 (4)	0.657 (5)	0.148*	
H13B	0.314 (12)	0.857 (4)	0.698 (5)	0.148*	

### Atomic displacement parameters (Å^2^)

**Table:**

	*U*^11^	*U*^22^	*U*^33^	*U*^12^	*U*^13^	*U*^23^
C1	0.0465 (14)	0.0255 (11)	0.0430 (13)	0.0029 (9)	0.0114 (11)	0.0018 (9)
C2	0.0433 (15)	0.0386 (13)	0.0572 (17)	0.0012 (11)	−0.0001 (12)	−0.0006 (12)
C3	0.0386 (14)	0.0371 (13)	0.072 (2)	−0.0061 (11)	0.0098 (13)	0.0020 (13)
C4	0.0540 (16)	0.0288 (11)	0.0487 (15)	0.0009 (11)	0.0189 (13)	−0.0001 (10)
C5	0.0567 (17)	0.0378 (13)	0.0515 (17)	−0.0028 (12)	−0.0034 (13)	0.0017 (12)
C6	0.0461 (15)	0.0360 (13)	0.0604 (17)	−0.0075 (11)	0.0012 (12)	−0.0022 (12)
C7	0.074 (2)	0.0380 (14)	0.0532 (17)	−0.0042 (13)	0.0280 (16)	0.0007 (12)
C8	0.0533 (16)	0.0303 (11)	0.0443 (14)	−0.0045 (11)	0.0166 (12)	0.0069 (10)
C9	0.0385 (14)	0.0394 (14)	0.0615 (17)	0.0006 (11)	0.0053 (12)	0.0073 (12)
C10	0.0426 (13)	0.0405 (13)	0.0516 (15)	−0.0040 (11)	−0.0003 (11)	−0.0008 (12)
C11	0.0439 (14)	0.0317 (11)	0.0450 (14)	−0.0026 (10)	0.0110 (11)	0.0057 (10)
C12	0.0395 (13)	0.0438 (13)	0.0489 (15)	−0.0009 (12)	0.0006 (11)	0.0020 (13)
C13	0.0526 (16)	0.0453 (14)	0.0476 (16)	−0.0092 (12)	0.0011 (12)	−0.0004 (12)
N1	0.0522 (14)	0.0327 (11)	0.0493 (14)	−0.0007 (10)	0.0109 (11)	0.0053 (10)
N2	0.0526 (16)	0.0430 (13)	0.0598 (17)	−0.0009 (11)	0.0102 (13)	−0.0071 (11)
C14	0.0373 (12)	0.0294 (10)	0.0354 (12)	−0.0008 (9)	0.0033 (10)	0.0028 (9)
C15	0.0399 (13)	0.0297 (10)	0.0399 (13)	−0.0018 (9)	0.0024 (10)	−0.0014 (10)
C16	0.0502 (15)	0.0355 (12)	0.0506 (16)	0.0006 (11)	0.0204 (12)	−0.0063 (11)
C17	0.0437 (14)	0.0350 (12)	0.0452 (14)	−0.0063 (10)	0.0155 (11)	−0.0015 (11)
C18	0.0416 (14)	0.0255 (10)	0.0373 (13)	−0.0028 (9)	0.0050 (10)	−0.0019 (9)
C19	0.0395 (13)	0.0295 (10)	0.0369 (13)	−0.0001 (9)	0.0076 (10)	−0.0003 (9)
C20	0.0408 (13)	0.0352 (12)	0.0378 (13)	−0.0018 (10)	0.0042 (10)	0.0072 (10)
O1	0.0584 (11)	0.0307 (9)	0.0610 (12)	−0.0109 (8)	0.0165 (9)	0.0016 (8)
O2	0.0503 (12)	0.0434 (10)	0.0609 (12)	−0.0058 (8)	0.0237 (9)	0.0028 (9)
O3	0.0632 (12)	0.0300 (9)	0.0572 (12)	−0.0089 (8)	0.0202 (10)	−0.0088 (8)
O4	0.0591 (12)	0.0426 (11)	0.0648 (13)	−0.0178 (9)	0.0169 (10)	−0.0127 (9)
O5	0.0722 (14)	0.0328 (9)	0.0542 (12)	0.0043 (9)	0.0013 (10)	0.0003 (8)
O6	0.0616 (12)	0.0444 (9)	0.0418 (10)	−0.0044 (9)	0.0053 (8)	−0.0081 (9)
S1	0.0465 (3)	0.0268 (2)	0.0410 (3)	−0.0056 (2)	0.0090 (2)	−0.0048 (2)
C21	0.0487 (14)	0.0309 (11)	0.0366 (12)	0.0026 (10)	0.0093 (11)	−0.0031 (10)
C22	0.0445 (14)	0.0435 (13)	0.0382 (13)	0.0094 (11)	0.0041 (11)	−0.0046 (11)
C23	0.0355 (13)	0.0548 (15)	0.0425 (14)	−0.0062 (11)	0.0078 (11)	−0.0064 (12)
C24	0.0403 (13)	0.0396 (12)	0.0415 (13)	−0.0027 (11)	0.0108 (10)	0.0017 (11)
C25	0.0403 (13)	0.0353 (12)	0.0352 (13)	0.0007 (10)	0.0063 (10)	−0.0040 (10)
C26	0.0375 (12)	0.0355 (11)	0.0398 (13)	−0.0009 (10)	0.0077 (10)	−0.0025 (10)
C27	0.0587 (16)	0.0359 (12)	0.0461 (15)	0.0022 (13)	0.0098 (12)	0.0003 (13)
O7	0.0983 (19)	0.0600 (15)	0.0541 (14)	−0.0140 (12)	−0.0090 (13)	0.0217 (11)
O8	0.0705 (14)	0.0522 (12)	0.0510 (13)	−0.0155 (10)	0.0072 (11)	0.0091 (10)
O9	0.0532 (12)	0.0598 (13)	0.0519 (12)	0.0058 (10)	−0.0041 (9)	0.0067 (10)
O10	0.0848 (17)	0.0520 (12)	0.0842 (17)	0.0241 (12)	0.0150 (14)	0.0013 (12)
O11	0.0452 (10)	0.0580 (12)	0.0537 (11)	−0.0025 (9)	0.0091 (8)	0.0180 (9)
O12	0.0511 (12)	0.0624 (13)	0.0609 (13)	−0.0159 (10)	−0.0087 (10)	0.0176 (10)
S2	0.0342 (3)	0.0355 (3)	0.0456 (3)	0.0013 (2)	0.0084 (2)	0.0066 (3)
O13	0.136 (3)	0.0565 (15)	0.112 (3)	0.0003 (17)	0.058 (2)	−0.0240 (16)

### Geometric parameters (Å, °)

**Table:**

C1—C6	1.368 (4)	C16—C17	1.371 (3)
C1—C2	1.373 (4)	C16—H16	0.9300
C1—N1	1.461 (3)	C17—C18	1.389 (3)
C2—C3	1.381 (4)	C17—H17	0.9300
C2—H2	0.9300	C18—C19	1.370 (3)
C3—C4	1.384 (4)	C18—S1	1.772 (2)
C3—H3	0.9300	C19—H19	0.9300
C4—C5	1.372 (4)	C20—O1	1.220 (3)
C4—C7	1.510 (4)	C20—O2	1.305 (3)
C5—C6	1.390 (4)	O2—H2D	0.80 (4)
C5—H5	0.9300	O3—H3A	0.86 (4)
C6—H6	0.9300	O4—S1	1.454 (2)
C7—C8	1.524 (4)	O5—S1	1.446 (2)
C7—H7A	0.9700	O6—S1	1.4490 (19)
C7—H7B	0.9700	O6—O13	3.064 (4)
C8—C13	1.377 (4)	C21—C26	1.388 (4)
C8—C9	1.386 (4)	C21—C22	1.396 (4)
C9—C10	1.378 (4)	C21—C27	1.484 (4)
C9—H9	0.9300	C22—O9	1.353 (3)
C10—C11	1.367 (4)	C22—C23	1.400 (4)
C10—H10	0.9300	C23—C24	1.375 (4)
C11—C12	1.376 (4)	C23—H23	0.9300
C11—N2	1.454 (3)	C24—C25	1.399 (4)
C12—C13	1.384 (4)	C24—H24	0.9300
C12—H12	0.9300	C25—C26	1.382 (3)
C13—H13	0.9300	C25—S2	1.763 (3)
N1—H1B	0.82 (4)	C26—H26	0.9300
N1—H1A	0.90 (4)	C27—O7	1.211 (4)
N1—H1C	0.99 (4)	C27—O8	1.311 (4)
N2—H2A	0.82 (4)	O8—H8A	0.87 (5)
N2—H2B	0.84 (4)	O9—H9A	0.86 (5)
N2—H2C	0.97 (4)	O10—S2	1.431 (2)
C14—C15	1.396 (3)	O11—S2	1.4526 (19)
C14—C19	1.405 (3)	O12—S2	1.439 (2)
C14—C20	1.487 (3)	O13—H13A	0.83 (8)
C15—O3	1.357 (3)	O13—H13B	0.89 (7)
C15—C16	1.384 (4)		
			
C6—C1—C2	121.0 (2)	C16—C15—C14	119.7 (2)
C6—C1—N1	119.4 (2)	C17—C16—C15	120.5 (2)
C2—C1—N1	119.5 (2)	C17—C16—H16	119.8
C1—C2—C3	119.0 (3)	C15—C16—H16	119.8
C1—C2—H2	120.5	C16—C17—C18	120.1 (2)
C3—C2—H2	120.5	C16—C17—H17	119.9
C2—C3—C4	121.4 (3)	C18—C17—H17	119.9
C2—C3—H3	119.3	C19—C18—C17	120.4 (2)
C4—C3—H3	119.3	C19—C18—S1	119.29 (18)
C5—C4—C3	118.3 (2)	C17—C18—S1	120.28 (18)
C5—C4—C7	120.0 (3)	C18—C19—C14	119.9 (2)
C3—C4—C7	121.6 (3)	C18—C19—H19	120.1
C4—C5—C6	121.1 (3)	C14—C19—H19	120.1
C4—C5—H5	119.5	O1—C20—O2	123.5 (2)
C6—C5—H5	119.5	O1—C20—C14	121.3 (2)
C1—C6—C5	119.2 (3)	O2—C20—C14	115.3 (2)
C1—C6—H6	120.4	C20—O2—H2D	106 (3)
C5—C6—H6	120.4	C15—O3—H3A	104 (3)
C4—C7—C8	110.9 (2)	S1—O6—H2B	111.3 (10)
C4—C7—H7A	109.5	S1—O6—O13	135.06 (12)
C8—C7—H7A	109.5	H2B—O6—O13	99.8 (10)
C4—C7—H7B	109.5	O5—S1—O6	111.97 (12)
C8—C7—H7B	109.5	O5—S1—O4	110.99 (13)
H7A—C7—H7B	108.1	O6—S1—O4	113.21 (12)
C13—C8—C9	118.4 (2)	O5—S1—C18	107.80 (12)
C13—C8—C7	121.2 (3)	O6—S1—C18	106.63 (12)
C9—C8—C7	120.4 (3)	O4—S1—C18	105.80 (11)
C10—C9—C8	121.2 (3)	C26—C21—C22	119.5 (2)
C10—C9—H9	119.4	C26—C21—C27	120.3 (2)
C8—C9—H9	119.4	C22—C21—C27	120.1 (2)
C11—C10—C9	119.2 (3)	O9—C22—C21	123.8 (2)
C11—C10—H10	120.4	O9—C22—C23	116.9 (2)
C9—C10—H10	120.4	C21—C22—C23	119.3 (2)
C10—C11—C12	121.2 (2)	C24—C23—C22	120.8 (2)
C10—C11—N2	119.1 (3)	C24—C23—H23	119.6
C12—C11—N2	119.7 (2)	C22—C23—H23	119.6
C11—C12—C13	118.9 (2)	C23—C24—C25	119.7 (2)
C11—C12—H12	120.6	C23—C24—H24	120.1
C13—C12—H12	120.6	C25—C24—H24	120.1
C8—C13—C12	121.2 (3)	C26—C25—C24	119.7 (2)
C8—C13—H13	119.4	C26—C25—S2	120.4 (2)
C12—C13—H13	119.4	C24—C25—S2	119.95 (19)
C1—N1—H1B	112 (2)	C25—C26—C21	120.9 (2)
C1—N1—H1A	114 (2)	C25—C26—H26	119.5
H1B—N1—H1A	114 (3)	C21—C26—H26	119.5
C1—N1—H1C	113.3 (19)	O7—C27—O8	123.6 (3)
H1B—N1—H1C	103 (3)	O7—C27—C21	122.4 (3)
H1A—N1—H1C	100 (3)	O8—C27—C21	114.0 (2)
C11—N2—H2A	114 (3)	C27—O8—H8A	112 (3)
C11—N2—H2B	113 (2)	C22—O9—H9A	109 (3)
H2A—N2—H2B	100 (4)	O10—S2—O12	112.51 (15)
C11—N2—H2C	119 (2)	O10—S2—O11	113.65 (14)
H2A—N2—H2C	107 (3)	O12—S2—O11	111.27 (13)
H2B—N2—H2C	102 (3)	O10—S2—C25	107.66 (14)
C15—C14—C19	119.3 (2)	O12—S2—C25	106.02 (12)
C15—C14—C20	119.7 (2)	O11—S2—C25	105.07 (11)
C19—C14—C20	121.0 (2)	O6—O13—H13A	127 (5)
O3—C15—C16	118.2 (2)	H13A—O13—H13B	106 (6)
O3—C15—C14	122.1 (2)		
			
C6—C1—C2—C3	0.1 (4)	C19—C14—C20—O1	−178.7 (3)
N1—C1—C2—C3	−179.4 (3)	C15—C14—C20—O2	−178.7 (2)
C1—C2—C3—C4	0.1 (4)	C19—C14—C20—O2	0.8 (4)
C2—C3—C4—C5	−0.9 (4)	H2B—O6—S1—O5	−2.9 (10)
C2—C3—C4—C7	176.9 (3)	O13—O6—S1—O5	−133.47 (18)
C3—C4—C5—C6	1.5 (4)	H2B—O6—S1—O4	−129.3 (10)
C7—C4—C5—C6	−176.3 (3)	O13—O6—S1—O4	100.15 (19)
C2—C1—C6—C5	0.5 (4)	H2B—O6—S1—C18	114.8 (10)
N1—C1—C6—C5	180.0 (3)	O13—O6—S1—C18	−15.8 (2)
C4—C5—C6—C1	−1.4 (4)	C19—C18—S1—O5	82.1 (2)
C5—C4—C7—C8	88.5 (3)	C17—C18—S1—O5	−99.1 (2)
C3—C4—C7—C8	−89.3 (3)	C19—C18—S1—O6	−38.3 (2)
C4—C7—C8—C13	−95.7 (3)	C17—C18—S1—O6	140.5 (2)
C4—C7—C8—C9	81.2 (3)	C19—C18—S1—O4	−159.1 (2)
C13—C8—C9—C10	−0.9 (4)	C17—C18—S1—O4	19.7 (3)
C7—C8—C9—C10	−177.9 (2)	C26—C21—C22—O9	−177.7 (2)
C8—C9—C10—C11	1.6 (4)	C27—C21—C22—O9	−1.6 (4)
C9—C10—C11—C12	−1.6 (4)	C26—C21—C22—C23	2.6 (4)
C9—C10—C11—N2	176.1 (2)	C27—C21—C22—C23	178.7 (2)
C10—C11—C12—C13	0.8 (4)	O9—C22—C23—C24	176.9 (2)
N2—C11—C12—C13	−176.8 (3)	C21—C22—C23—C24	−3.4 (4)
C9—C8—C13—C12	0.1 (4)	C22—C23—C24—C25	1.5 (4)
C7—C8—C13—C12	177.1 (2)	C23—C24—C25—C26	1.1 (4)
C11—C12—C13—C8	−0.1 (4)	C23—C24—C25—S2	−177.24 (19)
C19—C14—C15—O3	−175.8 (2)	C24—C25—C26—C21	−1.9 (4)
C20—C14—C15—O3	3.7 (4)	S2—C25—C26—C21	176.48 (18)
C19—C14—C15—C16	2.9 (4)	C22—C21—C26—C25	0.0 (4)
C20—C14—C15—C16	−177.6 (2)	C27—C21—C26—C25	−176.1 (2)
O3—C15—C16—C17	175.3 (3)	C26—C21—C27—O7	160.4 (3)
C14—C15—C16—C17	−3.5 (4)	C22—C21—C27—O7	−15.6 (4)
C15—C16—C17—C18	1.6 (4)	C26—C21—C27—O8	−17.9 (3)
C16—C17—C18—C19	0.8 (4)	C22—C21—C27—O8	166.1 (2)
C16—C17—C18—S1	−178.0 (2)	C26—C25—S2—O10	−101.6 (2)
C17—C18—C19—C14	−1.3 (4)	C24—C25—S2—O10	76.8 (2)
S1—C18—C19—C14	177.48 (19)	C26—C25—S2—O12	19.1 (2)
C15—C14—C19—C18	−0.5 (4)	C24—C25—S2—O12	−162.6 (2)
C20—C14—C19—C18	180.0 (2)	C26—C25—S2—O11	137.0 (2)
C15—C14—C20—O1	1.8 (4)	C24—C25—S2—O11	−44.7 (2)

### Hydrogen-bond geometry (Å, °)

**Table:**

*D*—H···*A*	*D*—H	H···*A*	*D*···*A*	*D*—H···*A*
N1—H1A···O4^i^	0.90 (4)	2.03 (4)	2.896 (4)	161 (3)
N1—H1B···O1^ii^	0.82 (4)	2.47 (3)	2.751 (3)	101 (3)
N1—H1B···O12	0.82 (4)	2.01 (4)	2.814 (4)	168 (3)
N1—H1C···O11^iii^	0.99 (4)	1.92 (4)	2.870 (3)	161 (3)
N2—H2A···O4^iii^	0.82 (4)	2.07 (4)	2.801 (4)	149 (3)
N2—H2C···O5	0.97 (4)	2.16 (4)	2.927 (4)	135 (3)
N2—H2B···O6	0.84 (4)	2.20 (4)	2.889 (4)	139 (3)
N2—H2C···O3^i^	0.97 (4)	2.19 (4)	2.940 (3)	134 (3)
O2—H2D···O11^iv^	0.80 (4)	1.91 (4)	2.688 (3)	164 (4)
O3—H3A···O1	0.86 (4)	1.77 (4)	2.569 (3)	153 (4)
O8—H8A···O13^v^	0.87 (5)	1.76 (5)	2.598 (4)	160 (4)
O9—H9A···O7	0.86 (5)	1.96 (4)	2.678 (3)	141 (4)
O9—H9A···O6^vi^	0.86 (5)	2.38 (4)	2.872 (3)	117 (3)
O13—H13A···O10^vii^	0.83 (8)	1.93 (8)	2.759 (4)	176 (7)
O13—H13B···O6	0.89 (7)	2.39 (7)	3.064 (4)	132 (6)
C2—H2···O5^viii^	0.93	2.54	3.452 (4)	168.
C6—H6···O12	0.93	2.48	3.210 (3)	136.
C12—H12···O9^ix^	0.93	2.55	3.428 (4)	158.
C16—H16···Cg^x^	0.93	2.85	3.727 (3)	157

Symmetry codes: (i) −*x*+1, *y*−1/2, −*z*+2; (ii) *x*, *y*−1, *z*; (iii) *x*+1, *y*, *z*; (iv) *x*+1, *y*+1, *z*; (v) −*x*+1, *y*−1/2, −*z*+1; (vi) −*x*, *y*−1/2, −*z*+1; (vii) *x*, *y*+1, *z*; (viii) −*x*+2, *y*−1/2, −*z*+2; (ix) −*x*, *y*+1/2, −*z*+1; (x) −*x*+1, *y*+1/2, −*z*+2.
